# Salivary IgA subtypes as novel disease biomarkers in systemic lupus erythematosus

**DOI:** 10.3389/fimmu.2023.1080154

**Published:** 2023-02-22

**Authors:** Sandra Romero-Ramírez, Víctor A. Sosa-Hernández, Rodrigo Cervantes-Díaz, Daniel A. Carrillo-Vázquez, David E. Meza-Sánchez, Carlos Núñez-Álvarez, Jiram Torres-Ruiz, Diana Gómez-Martín, José L. Maravillas-Montero

**Affiliations:** ^1^ Red de Apoyo a la Investigación, Universidad Nacional Autónoma de México e Instituto Nacional de Ciencias Médicas y Nutrición Salvador Zubirán, Mexico City, Mexico; ^2^ Facultad de Medicina, Universidad Nacional Autónoma de México, Mexico City, Mexico; ^3^ Departamento de Biomedicina Molecular, Centro de Investigación y de Estudios Avanzados del Instituto Politécnico Nacional, Mexico City, Mexico; ^4^ Departamento de Medicina Interna, Instituto Nacional de Ciencias Médicas y Nutrición Salvador Zubirán, Mexico City, Mexico; ^5^ Departamento de Inmunología y Reumatología, Instituto Nacional de Ciencias Médicas y Nutrición Salvador Zubirán, Mexico City, Mexico

**Keywords:** IgA, IgA1, IgA2, systemic lupus erythematosus, saliva

## Abstract

**Introduction:**

Immunoglobulin A (IgA) is the main antibody isotype in body fluids such as tears, intestinal mucous, colostrum, and saliva. There are two subtypes of IgA in humans: IgA1, mainly present in blood and mucosal sites, and IgA2, preferentially expressed in mucosal sites like the colon. In clinical practice, immunoglobulins are typically measured in venous or capillary blood; however, alternative samples, including saliva, are now being considered, given their non-invasive and easy collection nature. Several autoimmune diseases have been related to diverse abnormalities in oral mucosal immunity, such as rheumatoid arthritis, Sjogren’s syndrome, and systemic lupus erythematosus (SLE).

**Methods:**

We decided to evaluate the levels of both IgA subtypes in the saliva of SLE patients. A light chain capture-based ELISA measured specific IgA1 and IgA2 levels in a cohort of SLE patients compared with age and gender-matched healthy volunteers.

**Results:**

Surprisingly, our results indicated that in the saliva of SLE patients, total IgA and IgA1 subtype were significantly elevated; we also found that salivary IgA levels, particularly IgA2, positively correlate with anti-dsDNA IgG antibody titers. Strikingly, we also detected the presence of salivary anti-nucleosome IgA antibodies in SLE patients, a feature not previously reported elsewhere.

**Conclusions:**

According to our results and upon necessary validation, IgA characterization in saliva could represent a potentially helpful tool in the clinical care of SLE patients with the advantage of being a more straightforward, faster, and safer method than manipulating blood samples.

## Introduction

Systemic lupus erythematosus (SLE) is an autoimmune disease characterized by a deleterious immune response affecting several organs and tissues (1), challenging diagnostic and treatment approaches. SLE is typically defined by the presence of high titers of circulating autoantibodies (2) with abnormal numbers of T and B lymphocytes (3). Currently, the diagnostic criteria for SLE are predominantly based on the presence of clinical manifestations as well as the results of laboratory tests, such as low levels of C3/C4 complement components and the presence of anti-dsDNA or anti-Sm antibodies (4). However, these currently available markers for SLE diagnosis remain suboptimal regarding either their sensitivity or specificity (5). Consequently, more robust biomarkers for SLE are still needed to accurately diagnose patients, monitor disease progression and treatment effectiveness or predict future flares.

The common consensus in systemic lupus erythematosus (SLE) is that there is a general breakdown in lymphocyte tolerance. Consequently, autoreactive B cells are usually considered one of the central effector cell subsets responsible for maintaining inflammatory status in patients. Thus, B cells are considered among the main therapeutic targets of SLE treatment. Furthermore, B cell-targeted therapies have been applied to treat several autoimmune diseases such as pemphigus, multiple sclerosis, ANCA-associated vasculitis, and rheumatoid arthritis; therefore, hypogammaglobulinemia is recognized as a potential complication of these treatments (6, 7).

The European League Against Rheumatism (EULAR) and The American Academy of Asthma, Allergy, and Immunology (AAAAI) guidelines recommend assessing baseline immune function by testing serum immunoglobulins before or even after rituximab treatment in autoimmune disease (8–11). Nevertheless, only a small number of reports have emerged regarding aberrant immunoglobulin levels prior to starting B cell-depleting approaches in most autoimmune disorders; for example, polyclonal hypergammaglobulinemia is well documented in SLE. However, hypogammaglobulinemia has also been lupus-associated in patients exhibiting selective-isotype deficiencies (12).

Humoral immunity is essentially assessed by measuring titers of IgG-dominated serum antibodies, thus neglecting the contribution of IgA as the major immunoglobulin isotype in humans. To dimension that, it has been documented that the daily IgA production rates around 70 mg/kg of body weight exceed that of all other antibody isotypes combined (13–15). Moreover, IgA is the predominant antibody isotype in external secretions, including tears, intestinal mucous, colostrum, milk, and saliva (13–15), thus being well-known as the main mucosal immunoglobulin, playing a fundamental role as an immunological barrier that recognizes and excludes human pathogens (13–15). Beyond that, there are two human IgA subclasses: IgA1 and IgA2, both ubiquitously present but displaying a differential distribution in the body: IgA1 is dominant in serum or saliva, while IgA2 is most abundant in some other secretion fluids and the colon (16–20). In all body fluids, both IgA1 and IgA2 are mainly present as dimeric secretory IgA (SIgA) (15).

Numerous reports have documented a link between alterations in SIgA levels and inflammatory or autoimmune entities, nearly all using saliva for sampling purposes. In this way, patients with SLE, type I diabetes, oral lichen planus, overweight/obesity, oral submucous fibrosis, ankylosing spondylitis, rheumatoid arthritis, mixed connective tissue disease, and Sjögren’s syndrome have displayed significant higher total or antigen-specific salivary SIgA content than their healthy individuals’ counterparts (21–27). Although showing differences in SIgA titers, most of these studies have excluded salivary IgA1 and IgA2 subtypes assessment.

Information regarding the levels of salivary IgA subclasses in SLE is still lacking across existing reports. Most of the available efforts have been limited to the measurement of total IgA either in serum or saliva. Consequently, this study was undertaken to investigate the possible alterations in salivary IgA1 and IgA2 in a small cohort of patients with SLE and to characterize their association with disease features.

## Methods

### Patients and Healthy individuals

The study population comprised 14 healthy individuals and 38 SLE subjects divided into the following groups: 27 inactive lupus (SLEDAI ≤6), 11 with active disease (SLEDAI >6) were recruited from the department of Immunology and Rheumatology of the Instituto Nacional de Ciencias Médicas y Nutrición Salvador Zubirán. All SLE patients fulfilled ACR/SLICC 2012 classification criteria (28), and disease activity was addressed by the SLE disease activity index (SLEDAI). We excluded subjects with ongoing acute or chronic infections (i.e., HIV or viral hepatitis), pregnancy, and patients with a diagnosis of other concomitant autoimmune diseases except for antiphospholipid (aPL) syndrome. None of the study participants received any B cell-depleting or other biological therapies. This study was approved by the Institutional Ethics and Research Committees of the Instituto Nacional de Ciencias Médicas y Nutrición Salvador Zubirán (Ref. 2306). The patients/participants provided their written informed consent to participate prior to inclusion in the study. Demographic and Clinical characteristics of the study population are depicted in **Table 1**.

**Table 1:** Demographics, clinical and laboratory features of the cohort.

Features	Inactive SLE	Active SLE	P value
Gender - # (%)			
**Male**	3 (11)	2 (18)	
**Female**	24 (89)	9 (82)	
**Age in years-median**	32 (20-65)	34 (21-46)	0.31
Disease Activity-median			
**SLEDAI score (min-max)**	2 (0-4)	10 (6-25)	**<0.0001**
Laboratory Values- median (IQR)			
**Leukocytes (%)**	5.4 (4.4-6.2)	5.9 (2.7-7.1)	0.86
**Lymphocytes (%)**	29.3 (17.6-37.6)	13.6 (11-20)	**0.0095**
**Erytrocytes (%)**	4.7 (4.3-5.4)	4.0 (2.5-5.1)	0.3
**Monocytes (%)**	7.2 (6.4-11)	7.8 (6-13.9)	0.2
**Neutrophils (%)**	64.1 (53.4-69.6)	71.5 (67.1-78.5)	0.06
**Eosinophils (%)**	1.8 (0.95-3)	0.9 (0-1.9)	0.36
**Hemoglobin (g/dL)**	14.1 (13.5-14.9)	10.9 (7.4-15.6)	0.3
**Hematocrit (%)**	41.7 (40.6-47.9)	33.5 (22.6-46.4)	0.28
**Platelets (x10^9^/L)**	262 (219-274)	193.5 (135-304)	0.24
**Glucose**	89 (84-93)	92 (83-97)	0.87
**Aspartate aminotransferase (U/L)**	21 (15-24)	21 (19-24)	0.21
**Alanine aminotransferase (U/L)**	18 (11.7-25)	19 (8-25)	0.21
**C-reactive protein (mg/dL)**	0.14 (0.06-0.26)	0.5 (0.05-1.2)	**0.02**
**Creatinine (mg/dL)**	0.78 (0.62-0.81)	0.82 (0.6-0.9))	0.1
**C3 (mg/dL)**	105 (91-114)	92 (58-97)	0.053
**C4 (mg/dL)**	19 (11-23)	8 (8-14)	**0.0083**
**Anti-dsDNA (UI/mL)**	11.2 (6.5-139)	90.4 (11.2-212)	**0.043**
Type of disease activity - # (%)			
**Mucocutaneous**	1 (3.7)	2 (18.1)	
**Joint**	0 (0)	2 (18.1)	
**Serous**	0 (0)	1 (9)	
**Renal**	1 (3.7)	5 (45.4)	
**Hematological**	1 (3.7)	1 (9)	
**Nervous system**	0 (0)	0 (0)	
**Constitutional**	0 (0)	2 (18.1)	
Treatments - # (%)			
**Mycophenolate Mofetil**	10 (30)	3 (27.2)	
**Cyclophosphamide**	1 (3.7)	1 (9)	
**Prednisone**	14 (51.8)	8 (72.7)	
**Hydroxychloroquine**	17 (62.9)	4 (36.3)	
**Methotrexate**	7 (25.9)	1 (9)	
**Azathioprine**	3 (11.1)	3 (27.2)	

#, number of patients with the indicated feature. Bold text indicates a statistically significant differences with a p-value less than 0.05.

### Saliva sampling

All recruited subjects briefly rinsed their mouth with purified water before were asked to deposit a total volume of around 2 mL of unstimulated whole saliva by passive drooling (letting the saliva drop) into a sterile 15 mL polyethylene centrifuge tube. Immediately after collection, the tubes were transported to the laboratory into an ice bucket and were centrifuged for 15 min at 10,000 g and 4°C to remove debris and cells. The supernatants were then separated and added with 2x protease inhibitors (Pierce, Protease Inhibitor Tablets, Thermo Scientific) and finally stored at -70°C until used. All samples (from patients and healthy donors) were treated with this same procedure, not delaying more than 5 min from collection to centrifugation to minimize the risk of protein degradation.

### IgA1 and IgA2 quantification in saliva

Detection of IgA1 and IgA2 in saliva was performed by developing a sandwich ELISA previously reported (29). We used flat bottom microtiter plates (Thermo Scientific) that were coated overnight at 4°C with 1 µg/mL of anti-human Ig light chain antibody in 0.2 M Na_2_CO_3_/NaHCO_3_, pH: 9.4. Blocking was performed by using phosphate-buffered saline 1x containing 0.05% Tween-20 at room temperature for 2 h. Fifty microliters of saliva samples (in triplicate) and standard samples were pipetted into the microtiter wells and incubated for 2 h at 37°C. Among the various incubation steps, the wells were washed five times with phosphate-buffered saline 1x containing 0.05% Tween-20. Then, we added biotinylated anti-human IgA1 antibody (monoclonal mouse antibody, Abcam, Cat. Num. ab99796) diluted 1:2000 or biotinylated anti-human IgA2 antibody (monoclonal mouse antibody, Abcam, Cat. Num. ab128731) diluted 1:1000 in phosphate-buffered saline 1x containing 0.05% Tween-20 and incubated for 1 h at 37°C. After washing, we added Streptavidin-HRP diluted 1:5000 in phosphate-buffered saline 1x and incubated for 1h at 37°C. Then we washed six times with phosphate-buffered saline 1x containing 0.5% Tween-20. After a final wash, 50µL of the substrate solution, tetramethylbenzidine, was added, and the plates were incubated for 5min (to IgA1) and 10 min (to IgA2), and we used to stop enzyme reaction 50µL of 1N HCl. The absorbance was measured at 450nm using a microplate spectrophotometer. When indicated, the total IgA (tIgA) amount was obtained as the sum of measured IgA1 and IgA2 concentrations.

### Detection of salivary IgA anti-nucleosome and IgA anti-double stranded DNA antibodies

Salivary IgA anti-dsDNA antibodies and IgA anti-nucleosome were detected by ELISA kits QUANTA Lite ^®^ HA dsDNA and HA Nucleosome, respectively, employing an HRP anti-tIgA antibody for detection and following manufacturer instructions. The plates were then read at 450nm on a Bio-Rad xMark spectrophotometer.

### Statistical analysis

As indicated, differences between groups were analyzed using two-way ANOVA followed by Tukey’s *post hoc* test, Kruskal-Wallis tests followed by Dunn’s multiple comparisons tests, or Mann-Whitney U tests. The correlation of IgA1, IgA2, tIgA, laboratory, and clinical features, were evaluated by calculating the Spearman’s rank correlation coefficient. A *p* value less than 0.05 was considered statistically significant. ROC curve analysis was performed to distinguish IgA between healthy individuals and SLE patients or between inactive and active patients. All statistical analysis was performed using GraphPad Prism 9 software. Additionally, sensibility and specificity parameters for ROC curves were assessed using MedCalc 20.215 software.

## Results

### IgA1 is the predominant subtype in SLE saliva

**Figure 1:**
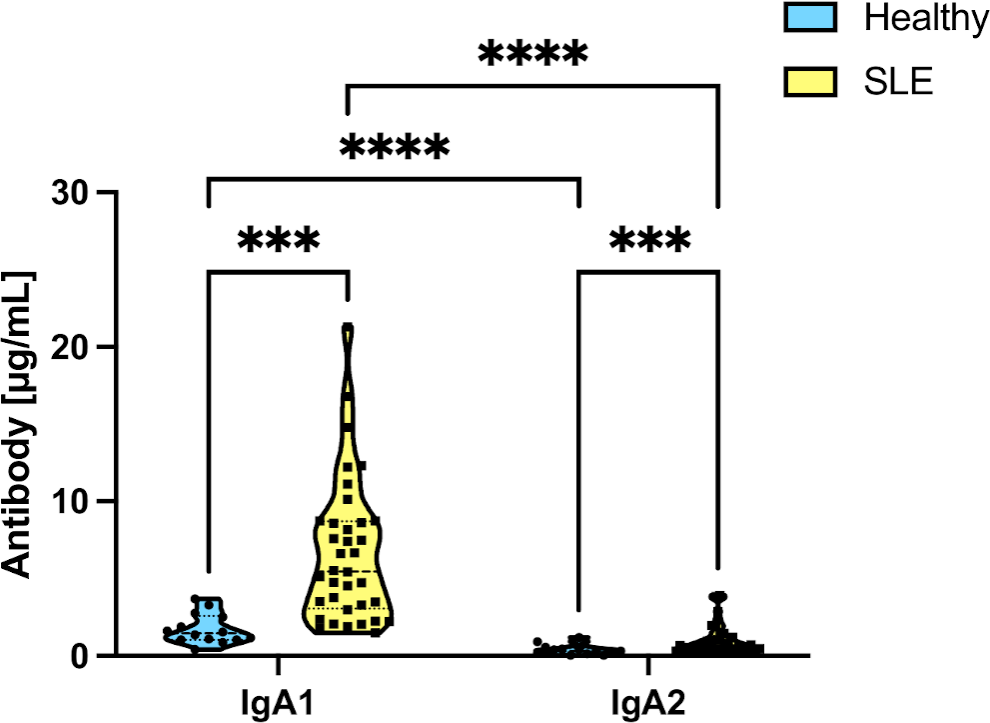
Differences in salivary IgA subtype levels in healthy individuals and SLE patients. Data were analyzed by an ordinary two-way ANOVA followed by Tukey’s *post hoc* test. ***p<0.001, ****p<0.0001.

We evaluated the levels of IgA antibodies in saliva, considering that the levels of the immunoglobulin subtypes vary in the different mucosal surfaces (30). A total of 52 individuals: 38 patients with SLE and 14 healthy individuals, were then evaluated to assess their concentration of SIgA salivary subtypes. As shown in **Figure 1**, the most dominant isotype in saliva was IgA1, but most importantly, healthy individuals showed significantly lower levels of IgA1 and IgA2 than patients with SLE.

### SLE patients exhibit higher levels of salivary SIgA subtypes than healthy controls

**Figure 2:**
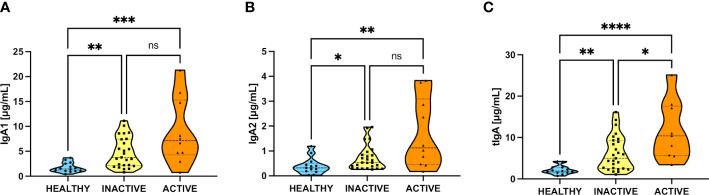
Increase of salivary IgA subtypes in inactive and active SLE patients. **(A)** Levels of IgA1 between healthy and inactive and active SLE patients. **(B)** Levels of IgA2 between healthy and inactive and active SLE patients. **(C)** Levels of tIgA (add of IgA1+IgA2) between healthy and inactive and active SLE patients. Data were assessed by Kruskall-Wallis tests followed by Dunn’s multiple comparisons tests. ns, not statistically significant, *p<0.05, **p<0.01, ***p<0.001, ****p<0.0001.

Given the clinical differences between inactive and active patients, we segregated our patients into these two groups as described in Methods. Then we analyzed their salivary IgA1, IgA2, and total IgA (tIgA) levels, as shown in **Figure 2**. Interestingly, both IgA subtypes and tIgA are significantly elevated in inactive and active patients compared to healthy individuals. Nonetheless, only increased tIgA levels were significantly different according to disease activity.

### Increasing concentrations in salivary subtypes of IgA correlate with titers of circulating anti-dsDNA antibodies in SLE

**Figure 3:**
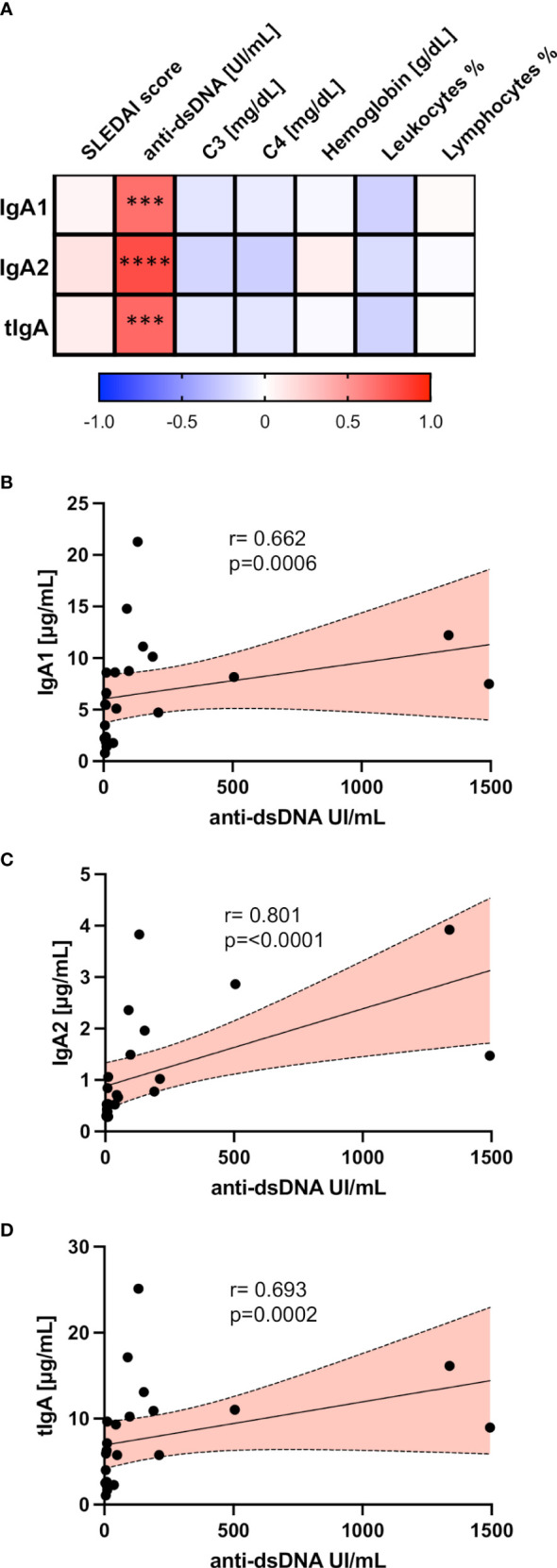
Salivary IgA1, IgA2, and tIgA correlated with different clinical variables in SLE. **(A)** Correlation matrix showing a graphical representation of calculations between IgA1, IgA2, tIgA, and laboratory and clinical variables of SLE patients. The underlying color scale indicates Spearman’s coefficient values. Correlation analysis between antibodies anti-dsDNA with salivary IgA1 **(B)**, salivary IgA2 **(C)**, and salivary tIgA **(D)**. Red slopes present a positive correlation. Correlations were assessed by calculating Spearman’s rank correlation coefficient (r) and *p* values (p) depicted. ***p<0.001, ****p<0.0001.

Trying to assess the importance of salivary IgA1 and IgA2 levels in SLE, we performed correlation analyses between these immunoglobulin isotype concentrations in saliva and different clinical and laboratory features of SLE activity (**Figure 3A**).

Remarkably, we only found positive and highly significant correlations between circulating anti-dsDNA with the salivary concentration of IgA1 (**Figure 3B**), IgA2 (**Figure 3C**), and tIgA (**Figure 3D**) in patients with SLE.

### Anti-nucleosome IgA autoantibodies are present and increased in the saliva of patients with SLE

Since the clinical diagnosis of SLE usually involves the detection of different blood-circulating autoantibodies, including levels of anti-dsDNA (double-stranded DNA) IgG and anti-nucleosome IgG in serum, we become interested in finding analog autoantibodies of IgA isotype in other types of samples such as saliva. Therefore, we evaluate the presence of salivary IgA-ANAs with nuclear or cytoplasmic staining patterns by indirect immunofluorescence. As shown in **Supplementary Fig. 1**, we detected typical autoimmune recognition patterns over HEp-2 cells when an anti-IgA was used as a detection antibody.

**Figure 4:**
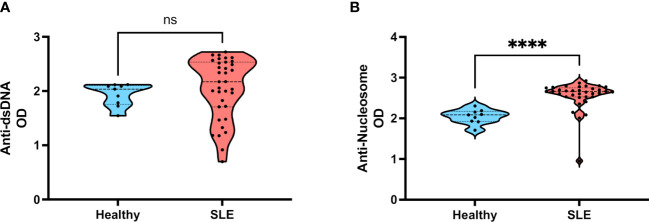
Levels of salivary IgA anti-dsDNA and salivary IgA anti-nucleosome in patients with SLE. **(A)** Salivary IgA anti-dsDNA in healthy individuals and SLE patients. **(B)** Salivary IgA anti-nucleosome in healthy individuals and SLE patients. Data were assessed by Mann-Whitney U tests. ns, not statistically significant, ****p<0.0001.

To address if salivary IgA fraction recognizes specific nuclear antigens like nucleosomes or dsDNA in our cohort, we performed commercially available ELISA assays employing an anti-human IgA detection antibody instead of the regular anti-human IgG used. As depicted by **Figure 4A**, we did not detect differences in salivary anti-dsDNA IgA antibodies between samples of healthy donors and SLE patients. In contrast, when IgA anti-nucleosome were measured, we found significantly higher titers of these autoantibodies in SLE patients compared with healthy individuals (**Figure 4B**). To verify if IgA anti-nuclear antigens were also present in the circulation of these patients, we measured these same autoantibodies in their serum samples. As expected, we found both anti-dsDNA IgA and anti-nucleosome IgA significantly elevated in SLE patients (**Supplementary Fig. 2**).

### IgA subtypes as potential salivary biomarkers of SLE

**Figure 5:**
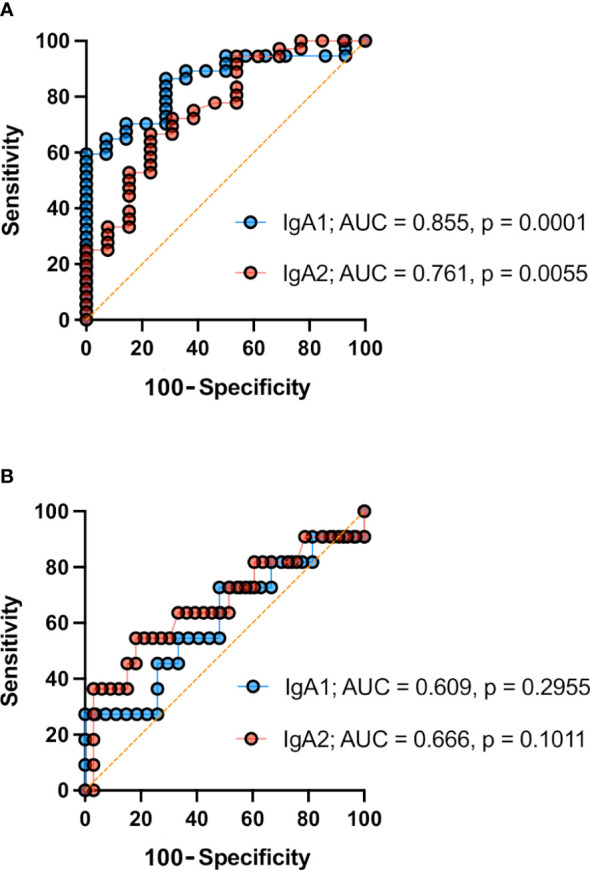
Receiver operator characteristics (ROC) curves of subtypes of salivary IgA for SLE discrimination and SLE activity prediction. **(A)** ROC curves of IgA subtypes for SLE discrimination. Data of n= 38 SLE patients and n=14 healthy individuals. **(B)** ROC curves of IgA subtypes for SLE activity prediction. Data of n= 27 inactive SLE patients and n=11 active SLE patients. Area under the curve (AUC) values and *p* values (p) are depicted.

Finally, to assess the usefulness of salivary IgA measurement as a potential SLE biomarker, we generated receiver operating characteristic (ROC) curves to determine the discriminative capacity of salivary IgA subtypes (IgA1 or IgA2) concentration in SLE patients vs. healthy donors (**Figure 5A**). We determined the corresponding areas under the curve (AUC), and we observed that IgA1 displayed an outstanding discriminative value (AUC of 0.855), but even IgA2 displayed a good value (AUC = 0.761).

As mentioned, SLE diagnosis relies on composite data from clinical manifestations and biomarkers such as serum autoantibodies (anti-dsDNA or anti-nucleosome) or complement (C3/C4) levels. Interestingly, when we compared the diagnostic robustness of salivary SIgA concentrations through their displayed AUC or sensitivity at 95% of specificity values with those from anti-dsDNA, anti-nucleosome, and complement levels in independent SLE cohorts reported previously (31, 32) as seen in **Supplementary Table 1**, these sensitivity/specificity values from SIgA are very similar to those displayed by traditional biomarkers, being IgA1 concentration the more robust.

Besides that, trying to distinguish between SLE inactive and active disease state contingent salivary IgA subclasses levels, we constructed an additional pair of ROC curves segregating these groups of patients. As shown in (**Figure 5B**) , the discriminative capacity of both IgA subtypes in saliva was satisfactory but lower than the diagnostic approach, according to their displayed AUC values (above 0.6 in both cases).

## Discussion

Humans generate about 1.5 L of saliva, which is secreted by the three major types of salivary glands (parotid, submandibular, and sublingual glands) and the minor salivary glands. Among the most recognized components of this fluid, secretory IgA constitutes the predominant immunoglobulin isotype in this and other body secretions.

Since IgA is also the predominant antibody isotype produced by the human body (33), it possesses an essential role in health and several diseases. Interestingly, increases in serum IgA and IgA autoantibodies have been reported in different autoimmune disorders like Sjogren’s syndrome, rheumatoid arthritis, IgA nephropathy, inflammatory bowel disease, and SLE (34–37).

On the other hand, the evidence on salivary IgA in those autoimmune diseases is limited and mostly inconclusive (34–37). Most studies are focused on reporting individuals with low IgA levels since IgA deficiency’s mortality and morbidity rates correlate with SLE activity, mainly due to recurrent infections in these patients (38). Conversely, only one previous report informs about significantly higher levels of salivary (total) IgA in patients with SLE compared to healthy individuals (39), a reason why we wanted to go further with this observation evaluating the IgA subclasses.

Beyond total IgA fraction, this antibody isotype consists of two subclasses in humans where their proportions vary depending on the mucosal site: in saliva, we usually detect around 60% of IgA1 and close to 40% of IgA2 in healthy individuals (30); however, there was no previous data on salivary IgA subtypes in autoimmune diseases such as SLE.

Interestingly, we found that both salivary IgA1 and IgA2 are elevated in SLE patients compared to healthy individuals, being IgA1 levels significantly higher than those from IgA2. Besides that, when the SLE group was segregated into inactive and active patients, we observed a clear trend towards increased IgA subtype levels in active SLE individuals. Although these elevations did not exhibit significant differences when each IgA subtype was evaluated, the analysis of the levels of total IgA (tIgA=IgA1+IgA2) displayed a significant increase according to global disease activity, an observation that was not previously reported. Accordingly, increases in the antibody fraction (IgA+IgG) in the saliva of lupus patients were observed in a previous study (39), but it does not refer to any individual subtypes’ characterization. Regarding subtypes, the only prior published evidence comes from Roos Ljungberg et al., which found salivary IgA1 and IgA2 anti-citrullinated protein antibodies in rheumatoid arthritis patients (26). The authors found a strong association between salivary IgA and disease activity, even better than serum IgA, suggesting effector mechanisms in this disease pathogenesis due to oral mucosal immune responses to citrullinated proteins (26). However, the significance of altered salivary IgA subclasses was not yet elucidated in this or other autoimmune diseases.

Our results suggest that increased salivary IgA could be associated with disease activity in SLE. Interestingly, observing the absolute amounts of each antibody subclass, and although IgA2 is clearly elevated in both groups of patients, it becomes evident that the most dramatic increase is in the IgA1 fraction that displays a mean value below 2 μg/mL in healthy controls and close to 10 μg/mL in active SLE patients. This last observation is remarkable since, in the oral cavity, IgA1 would be prone to degradation mediated by bacterial proteases, given its particular structure with a larger hinge region (40, 41). This rise in salivary IgA1 concentrations must result from an overproduction of this antibody that could probably be related to the increments in systemic IL-10 levels as previously reported in lupus patients (42, 43). IL-10 is an anti-inflammatory cytokine that promotes B cell responses and plays a pathogenic role in SLE (44). Beyond that, serum levels of this cytokine have been reported to correlate with lupus disease activity (45). Furthermore, it has been proposed that the class switch to IgA1 is mediated by IL-10 and TGF-β (46, 47). As IL-10 was also previously reported elevated in the saliva of SLE patients (48), the dysregulation exerted by that cytokine in the oral microenvironment could be a key element that supports our data.

Interestingly, both subclasses and the total amount of IgA showed robust and highly significant positive correlations with the circulating anti-dsDNA autoantibodies (defined as IgG isotype in serum). Anti-dsDNA antibodies represent a hallmark of SLE and constitute an effective parameter for diagnosing and classifying patients. Additionally, their fluctuating titers during the progression of the disease reflect its activity in many patients and even may predict disease relapse (49). Still limited by our cohort size, these strong correlation values raise the feasibility of employing salivary IgA measuring, either total or subtypes, as a surrogate marker of SLE activity. This possibility needs further exploration in more significant and prospective cohorts.

So far, we have discussed changes in the amount of IgA regardless of its specificity. Thus, with these salivary antibodies correlating with a well-established biomarker as serum IgG anti-dsDNA, we determined if saliva could contain these and other IgA-isotype anti-nuclear antibodies (ANAs). So, beyond the qualitative findings depicted in our autoreactive IgA detection by immunofluorescence employing HEp-2 cells, we decided to perform a semi-quantification of specific salivary IgA ANAs. Surprisingly, we could not find any difference regarding IgA anti-dsDNA. However, we detected a highly significant increase of IgA anti-nucleosome antibody titers in the saliva of patients with SLE versus healthy individuals. To demonstrate that these IgA autoantibodies were also present in our patient’s blood, we measured their levels with the same approach in serum samples. In this regard, we found that circulating IgA anti-dsDNA or IgA anti-nucleosome levels were highly increased in SLE patients, as expected, since they have been reported as elevated previously (50, 51).

It is important to clarify that the method used to quantify these autoantibodies is limited by the unavailability of IgA standards, thus not making it possible to measure them beyond a semi-quantitative approach. Besides that, saliva constitution (in contrast with the serum used to develop and validate the commercially available tests employed here) could interfere with the adequate determination of these autoantibodies and partially explain why our measurements in this fluid seem to present such a high fluorescence background. Besides this problem, which requires an independent standardization of a new ELISA method for saliva, the significantly increased levels of salivary IgA anti-nucleosome antibodies are still of great interest. Several reports mention those anti-nucleosome antibodies as a disease activity marker in patients with SLE (52) and even correlate better than other conventional biomarkers (C3/C4 or anti-dsDNA) with SLE disease activity over time (53), making of the measurement of salivary IgA anti-nucleosome an attractive possibility for the routine monitoring of SLE patients in clinical practice due to the easy access and availability of the type of sample contingent on the validation of these observations in larger/multicenter cohorts and the development of specific antibody tests for this purpose.

To gain insight into the potential of IgA antibodies as diagnostic biomarkers in SLE, we constructed ROC curves for IgA subtypes in the saliva of these patients. Our results indicate outstanding discriminative values for IgA1, and a lower but still good for IgA2 when distinguishing patients with SLE from healthy individuals.

As depicted, the sensitivity/specificity values given by measuring IgA isotypes in saliva regarding SLE diagnostic are comparable to those exhibited by other clinically relevant markers such as serum anti-dsDNA, anti-nucleosome, or complement levels in independent cohorts. This observation makes these secretory antibodies (particularly salivary IgA1 concentration) appealing candidates to be included as other criteria in SLE diagnostic current approaches upon prior validation in a larger multiethnic cohort.

Interestingly, when we performed the same analysis for assessing salivary IgA subtypes’ predictive ability when distinguishing inactive from active disease states, we could only obtain satisfactory AUC values for both antibody subtypes concentrations, making these two variables not as robust for activity discrimination. However, it becomes evident that including more patients, particularly those with active disease, could improve predictive values.

Again, as the measurement of these immunoglobulins supports the discrimination of SLE patients, their potential employment as a clinically useful biomarker for this disease becomes evident. One more time, our study remains limited due to the cross-sectional nature of our approaches; hence, we propose to conduct longitudinal monitoring of SLE patients to assess and/or validate the utility of IgA subtypes as new clinical biomarkers. Beyond that, these measurements should also be performed considering their predictive potential for different SLE implications, such as different types of flares.

Finally, as SLE patients can be treated with B cell-depleting biological therapeutics that consequently could diminish immunoglobulin levels, our proposed measurement of SIgA towards establishing a useful biomarker would be limited. The potential development of hypogammaglobulinemia is among the main concerns after administering biological treatments for SLE; for example, one of the most common drugs of this type employed for lupus treatment, rituximab, depletes CD20^+^ peripheral B cells for an average of 6-12 months, including naive and unswitched B cells, both of which are direct precursors for IgM production, thus further reducing the circulating levels of this isotype (54).

Different studies have informed that rituximab administration apparently does not affect the circulating IgA baseline levels in patients with SLE in the long term (55, 56). Conversely, other reports indicate only a slight initial decrease of IgA median levels that started recovering as early as two months after rituximab (54), a decrease that becomes significant upon cumulative cycles of treatment but only in a small percentage (around 3%) of patients (56). Supporting these data, it has previously been described that circulating IgA^+^ plasmablasts can persist early after rituximab, suggesting resistance to depletion of switched IgA+ precursor B cells, likely in the mucosal microenvironment and/or due to an early replenishment (57).

Currently, there is little information about secretory IgA levels upon biological treatment administration in contexts of autoimmune disease. However, one case report (58) indicates that rituximab treatment leads to an unexpected increase in the percentage of IgA^+^ plasmablasts in parotid salivary glands, compared with a biopsy before treatment.

All the previously mentioned data support the idea that biological treatments do not significantly affect the levels of IgA, even the secretory one in saliva; however, this hypothesis needs to be corroborated, including these treated patients in an independent study.

In conclusion, as the saliva sample is easily accessible and non-invasive for the patients, its IgA measurement emerges as an attractive alternative to being proposed as a novel biomarker of SLE. Given our results, salivary IgA subtypes correlate with specific autoantibodies related to disease diagnosis or activity but also may allow us to differentiate healthy individuals and SLE patients through the measurement of total salivary IgA or individual salivary subclasses, independently of antibody specificity.

## Data availability statement

The raw data supporting the conclusions of this article will be made available by the authors, without undue reservation.

## Ethics statement

The studies involving human participants were reviewed and approved by Institutional Ethics and Research Committees of the Instituto Nacional de Ciencias Médicas y Nutrición Salvador Zubirán (Ref. 2306). The patients/participants provided their written informed consent to participate in this study.

## Author contributions

SR-R, VS-H, and RC-D contributed to the design and performance of experiments, analysis, and interpretation of data. SR-R, VS-H, RC-D, DM-S, and CN-A performed experiments and analyzed data. DC-V and JT-R assisted in the processing and preservation of patient samples, collected patient data, and generated and organized our clinical database. JT-R and DG-M assisted in writing and editing the manuscript. SR-R, DG-M, and JM-M designed and performed experiments, supervised general work, wrote, and edited the manuscript. All authors contributed to the article and approved the submitted version.
