# Supplementary material for: Salivary IgA subtypes as novel disease biomarkers in systemic lupus erythematosus
Source: Front Immunol. 2023 Feb 22;14:1080154. doi: 10.3389/fimmu.2023.1080154 (PMC9992540; doi:10.3389/fimmu.2023.1080154)
Supplement: Supplementary file 1 [file DataSheet_1.pdf]

Supplementary Material

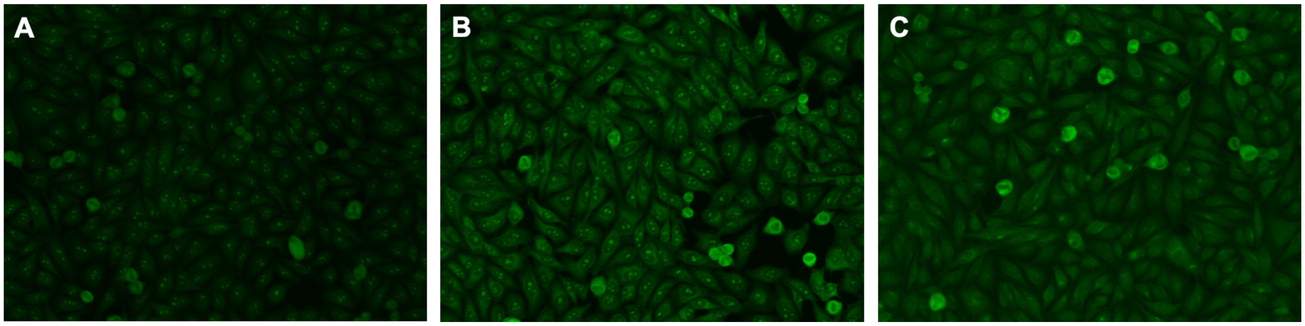

Supplementary Figure 1. Examples of IgA ANAs patterns contained in the saliva of SLE patients. Hep-2 indirect immunofluorescences employing an anti IgA as detection antibody. A. Nuclear-nucleolar, B. Nuclear-nucleolar and cytoplasmic. C. Cytoplasmic and polar/Golgi-like. 40x in all fields.

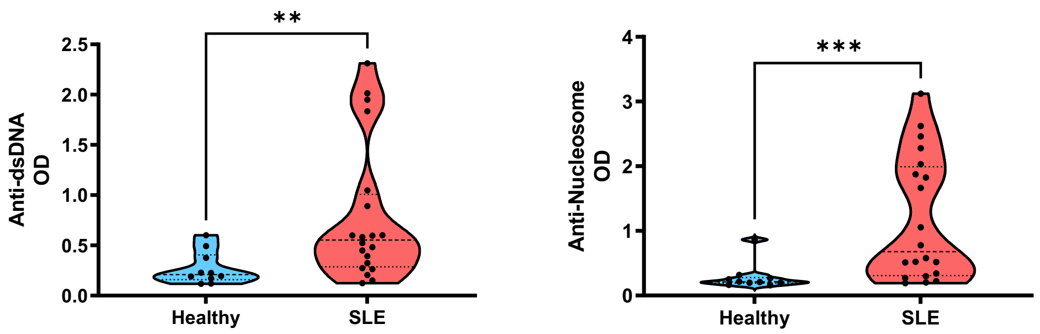

Supplementary Figure 2. Levels of serum IgA anti-dsDNA and serum IgA anti-nucleosome in patients with SLE. A. Serum IgA anti-dsDNA in healthy individuals and SLE patients. B. Serum IgA anti-nucleosome in healthy individuals and SLE patients. Data were assessed from n= 20 SLE patients and n=10 healthy individuals by Mann-Whitney U tests. **p<0.01, ***p<0.001.

Supplementary Table 1. Sensitivity/specificity values for salivary IgA subtypes and commonly used diagnostic markers in SLE.

Marker	AUC	Sensitivity at 95% specificity	Reference
Salivary IgA1	0.855	59.46%	This work
Salivary IgA2	0.761	26.32%	This work
Serum anti-dsDNA	0.800	41.20%	31
Serum anti-nucleosome	0.704	26.70%	31
C3/C4	0.730/0.720	44.00%	32
